# Exploring Van Gogh Syndrome: A Case Report on Schizoaffective Disorder and Self-Harm

**DOI:** 10.1155/crps/9655675

**Published:** 2025-10-16

**Authors:** Shereen Aly, Ahmad Ayman Hasanoglu, Oraib Abdallah

**Affiliations:** ^1^Psychiatry Department, Mental Health Services, Hamad Medical Corporation, Doha, Qatar; ^2^Pharmacy Department, Mental Health Services, Hamad Medical Corporation, Doha, Qatar

## Abstract

Deliberate self-harm (DSH) is defined as the intentional infliction of physical injury on oneself without the intent to end one's life. Common behaviors associated with DSH include cutting with a knife or razor, scratching or hitting oneself, and intentional drug overdose. Additionally, these behaviors may encompass restricting food intake and engaging in other risk-taking activities, such as driving at high speeds and participating in unprotected sexual activities. DSH is a strong indicator of suicide risk, particularly in individuals with schizophrenia. The death of Vincent Van Gogh on July 29, 1890, at the age of 37, following a firearm-related suicide attempt, is a compelling example. This occurred a year after his infamous act of self-inflicted ear mutilation, underscoring the increased suicide risk among individuals with a history of significant self-mutilation. We report a similar case of a patient who presented with superficial new cuts on a broken index finger of the left upper limb. Further assessment revealed schizoaffective disorder, which required close monitoring and management. There is a need to enhance the knowledge of identifying those at an elevated risk of self-harm and, whenever feasible, to implement appropriate harm-reduction strategies.

## 1. Introduction

Vincent Van Gogh (1853–1890) is one of the most important painters in our history [1]. He created a considerable number of masterpieces in just one decade of devoting himself to art. His productivity is even more remarkable when considered in the context of his debilitating illness [2].

**Figure 1:**
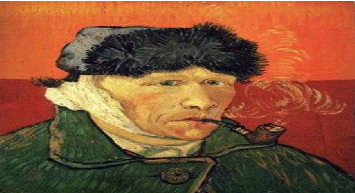
Famous Van Gogh self-portrait depicting his mental state after a brutal argument with painter Paul Gauguin, after which he cut off his right earlobe. He is seen smoking a pipe with a bandaged ear.

The perception of Van Gogh as a “mad genius” is largely shaped by the way he represented himself through his artwork [3]. In his Self-Portrait with a Bandaged Ear (1889), he dwells on the wound he inflicted on himself when he sliced his ear with a razor blade in Arles in December 1888 and presented the resulting chunk of severed flesh to a local prostitute. He shows us his maimed face, but it gazes back at us with the blue eyes of a visionary. (Figure 1) This is not an objective record of misfortune, but in its hypnotic intensity, a portrait of the artist both martyred and liberated by madness [4].

A recently discovered letter from Dr. Felix Rey, who treated Van Gogh's wound, explains the full horror beneath the bandage in his 1889 self-portrait. This confirms that Van Gogh did not just slice off his earlobe, as had been widely assumed, but sliced off his entire left ear. This makes it clearer than ever what an extreme act of self-harm it was and how it foreshadowed his death by suicide [4].

This incident has led to what has been called the Van Gogh syndrome, which has now become a synonym for nonsuicidal self-injury (NSSI), where participants intentionally and repetitively inflict injuries on their bodies without suicidal intention and are not socially sanctioned to do so. These injuries are not meant to cause lethal harm and range from biting, scratching, and cutting to serious acts such as mutilating reproductive parts [5, 6]. This behavior is common among adolescents and women psychiatric patients. Bipolar disorder, drug abuse, Munchausen's syndrome, and metabolic syndromes such as Lesch–Nyhan syndrome are often associated with this disorder [7]. In individuals with psychosis, it is typically due to a delusional belief or in response to imperative auditory hallucinations or a made phenomenon [8]. While “Van Gogh syndrome” is not a formally recognized diagnostic entity in major classification systems, as it is not listed in DSM-5 or ICD-10, several cases do use this term descriptively for severe self-mutilation in psychotic disorders [1, 2]. There have been several cautions to use the term, and “syndrome” is mentioned here descriptively, but should not be a primary diagnosis.

Although major self-mutilation (MSM) is extremely uncommon, early treatment of psychotic illness may reduce its incidence [9]. Reflecting on Van Gogh's tragic story helps us understand the complexities of psychiatric disorders and their manifestations. It is within this context that we present a contemporary case of self-mutilation in a schizoaffective patient, illustrating the enduring challenge of understanding and treating such conditions.

## 2. Case Presentation

**Figure 2:**
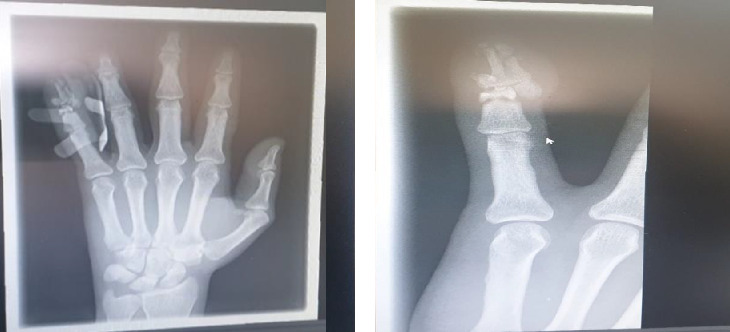
X-ray of (a) the left hand and (b) the left little finger showed that there is a comminuted fracture involving the middle and distal phalanx of the left little finger.

We report the case of a South Asian male patient aged 29 years old from the psychiatry department at Hamad Hospital, Doha, Qatar, who worked as an electrician, lived with coworkers, and had been in the country for 2 years. In January 2021, he presented to the emergency department with an alleged history of a left little finger injury caused by a brick that fell on it while he was working. Local examination revealed a laceration wound on the radial side of the distal phalanx of the left fifth finger, swelling, and limited movement of the left little finger. The patient could not flex his fingers, but his sensation was intact. The left-hand radiograph shows a comminuted fracture involving the middle and distal phalanx of the left little finger (Figure 2). A plastic surgery team was consulted, and the operation was performed under local anesthesia for closed reduction using a single K-wire fixation, and a volar slap was applied. Radiography of the left hand and left little finger revealed a comminuted fracture involving the middle and distal phalanx of the left little finger (Figure 2). The patient was then discharged and given an outpatient appointment in addition to home medications that included antibiotics and analgesics.

One day after discharge, the patient presented to the ED with superficial new cuts on the broken index finger of her left upper limb. The patient complained of low mood. He mentioned that he had depression 2 years earlier. He was on medication before arriving in Qatar, which he discontinued because of side effects; however, he did not elaborate on the medication or side effects. Psychiatric consultation was sought, which revealed that he tried to inflict injury with a knife on the same hand with the fracture, saying, “ If you love someone and you want to impress him, you will cut your finger.” When asked about whom you want to impress and love, he said, “The creator.” He said, “Allah gave him everything, but he did not do anything to thank Allah, so he offered his finger as a sacrifice.” He was in the kitchen when he picked up a knife and pressed it against his fingers. He stopped because he started feeling pain as the knife became dull. He was looking for another knife. He then went out to the main road, used two marble stones, one below his finger, and hit the other. A man saw him and called for an ambulance. He was not confident in talking to others about what had happened. He had been hearing the voices of people for 15 days before admission, discussing among themselves and telling him to sacrifice a body part, saying, “You only have little time, and you have a lot of sins, so you must do something for Allah.” Moreover, “He does not want to cut other fingers because it is wrong; cutting his small finger is also wrong, but he will do it for unknown reasons; he feels something is controlling him to do so, but he does not know who or why.” He stated that although God did not order him to cut his finger, he had guided him to do so. When asked about suicide or self-ideation, he reported: “I am not sure if I will do it again, I might do it again, only God can prevent me.”

Upon further assessment, he reported that he had a low mood, excessive thinking, anhedonia, decreased appetite, weight loss, and decreased energy in the last 4 months, which worsened with time. Four days before harming himself, he became afraid of all other people and stayed in his room, and his appetite decreased. This happened because he was overthinking due to financial problems in his country. He reported that he had not slept well for a few days before admission. The patient had a past psychiatric history of low mood; it had happened twice before in his home country; the first time was in 2010, when he used Ganja to reduce his anxiety. The doctors performed gastric lavage on the patient. There were no ideas, such as cutting his finger, only fear, overthinking, and isolation. Two years ago, his family took him to see a psychiatrist. He stated that he took the prescribed medications for 3 months and then stopped because he was feeling better. The patient denied any family history of mental illness or substance abuse.

There was no family history of self-harm or self-mutilation. The patient's personal history revealed that he was born at full term via normal delivery with appropriate developmental milestones, and his childhood and adolescent periods were unremarkable. He had no history of abuse or neglect in childhood. He attended college but later dropped out of it.

Local examination revealed superficial new cuts on the index finger of the left upper limb that did not require suturing; glue was sufficient to close the wound. There were sutures in the same finger, which were performed 2 days before; the patient had no limitation of movement of the digit.

The patient was conscious, with a Glasgow Coma Scale (GCS) score of 15/15 and normal vital signs. No pain was noted, with the numeric pain rating scale (NPRS) score being zero, and he had an unremarkable systemic examination.

Routine investigations, including CBC, liver and renal functions, electrolytes, TSH, T4, T3, urine screening test, and CT scan, were all unremarkable.

**Figure 3:**
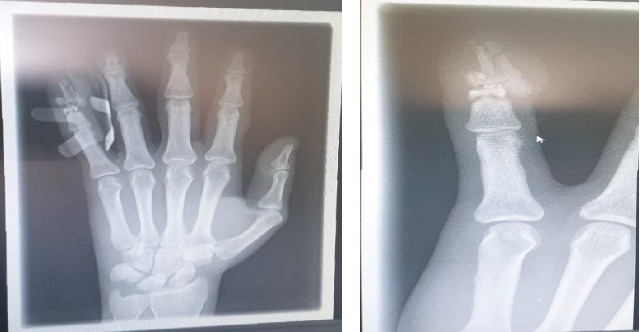
X-ray hand left findings: (a) finer bony details are obscured by the plaster slab and (b) an internal fixation pin is noted in the left little finger for fractures of the middle and distal phalanges.

The left hand radiograph shows finer bony details that were obscured by the plaster slab. An internal fixation pin was noted in the left little finger for middle and distal phalanx fractures (Figure 3).

The psychosexual and forensic histories were unremarkable. The patient denied drug and alcohol abuse. The premorbid personality of the patient's mood was predominantly depression and excessive worrying, and he had interests in music and going out with friends. No borderline personality or impulsive traits were observed.

On mental status examination, the patient's general appearance and behavior were of a middle-aged man, with his left hand in a cast, well-kempt, poor eye contact, smiling inappropriately, shy, and with a normal posture, and he displayed an uncooperative attitude. His speech was monotonous, with long pauses sometimes between the answers. The speech was sometimes irrelevant to the task. The effect was blunt with a restricted range, subjective mood was depressed, and there was decreased reactivity and communicability. The patient reported a slowing of thoughts, but no formal thought disorder was detected during serial mental examinations. Delusions of control, religious delusions, delusions of guilt, and second- and third-person auditory hallucinations were important. He reported that he had sins to erase and that God should forgive him. He felt that something was controlling him to cut his finger, but he did not know who or why, in response to the voices of his friends and family talking to each other and ordering him to do so as a sacrifice for God. As a person, he should give all his belongings to God, not just his body. His judgment and insight were impaired.  Table 1 summarizes MSE.

The Brief Psychiatric Rating Scale [10] score was 82/128, indicating the presence of psychosis. Hamilton Depression Rating Scale score was 20/52, indicating depression, positive and negative symptom scale (PANSS) score was P 25, N 24, G 68. A provisional diagnosis of schizoaffective disorder was made after serial mental status examinations and the presence of first-rank and depressive symptoms using the Diagnostic and Statistical Manual of Mental Disorders (DSM–5).

The patient was started on olanzapine 5 mg, which was gradually increased to 20 mg over 17 days, after which the patient no longer heard any voices, had no more thoughts about removing his finger, and his mood was euthymic. Local wound management with antibiotics and debridement continued during his hospital stay. The patient was discharged, and a follow-up appointment was scheduled at an outpatient clinic.

## 3. Discussion

Deliberate self-harm (DSH) encompasses a wide range of behaviors that cause self-injury, including suicidal and nonsuicidal actions. NSSI involves deliberately harming one's body without the intention of ending one's life. Most definitions emphasize immediate and direct harm to body tissue, excluding broader behaviors such as medication misuse and restrictive eating. Conversely, suicidal self-injurious behavior is characterized by the presence of a desire to die, such as in attempted or completed suicide [11].

NSSI has emerged as a distinctive characteristic observed in numerous clinical populations in recent years. Notably, schizophrenia is one of the most prevalent psychiatric disorders associated with NSSI [4]. MSM, a subset of NSSI, is a rare but potentially catastrophic complication associated with severe mental illnesses. Most individuals engaging in NSSI exhibit symptoms of psychotic disorders within the schizophrenia spectrum disorder (SSD) [5]. MSM manifests primarily in three forms: ocular, genital, and limb. Patients who have undertaken extreme acts, such as eye removal or limb amputation, are invariably in a psychotic state, with approximately three-quarters of those who inflict genital injuries falling into the same category [9].

Caroll et al. reported that for every 25 patients presenting to the hospital for self-harm, one will kill themselves in the next 5 years [12]. The mortality rate among a large group of individuals who attempted suicide and were monitored afterward was 3.3 times higher than anticipated. Over extended follow-up periods, the overall death rates were 1.5% within the first 2 years, 2.0% within the first 3 years, and 2.3% within the first 5 years. By the end of the eighth year, 2.8% of the patients had died by suicide or likely suicide, with the suicide death rate being 26.9 times the expected rate. The risk of suicide was most pronounced during the initial 3 years, particularly within the first 6 months following the attempt. Factors linked to an increased risk of suicide at the time of the attempts included being male, older age (in females), having a psychiatric disorder (notably schizophrenia), prolonged use of hypnotics, poor physical health, and repeated attempts to commit suicide [13].

A longitudinal study involving 11,583 patients who engaged in DSH and were admitted to a general hospital over two decades was conducted to assess the mortality risk from various causes over a follow-up period ranging from 3 to 23 years. The incidence of suicide was found to be 17 times higher than anticipated, whereas deaths from undetermined causes and accidental poisoning occurred 15 times more frequently than expected [14].

A Danish study conducted over a decade followed patients who were admitted to a poisoning treatment center in 1980 after attempting suicide. The study aimed to describe mortality rates due to suicide and other causes, as well as to identify factors that could predict these outcomes. During the 10-year follow-up, 306 patients died, 103 of whom had died by suicide [15].

The National Confidential Inquiry into suicides in England and Wales revealed that one in four suicides involved contact with mental health services in the year preceding death. Of the 219 individuals who eventually died by suicide, 85 (39%) had visited an accident and emergency department within the year prior to their death, with 15% of these visits related to nonfatal self-harm [16].

A study conducted by Mork et al. [17] revealed that the lifetime prevalence of NSSI episodes was as high as 48.5% among a sample comprising both inpatients and outpatients diagnosed with SSDs, including schizophrenia, schizoaffective disorder, and schizophreniform disorder. While self-injurious behavior can be associated with various disorders, including conduct disorder, borderline personality disorder, factitious disorder, and malingering, the severity of trauma witnessed in these instances is particularly noteworthy.

Furthermore, a history of prior self-harm, regardless of its severity, was found to be linked to subsequent self-injurious events, especially among individuals diagnosed with schizophrenia. Additionally, many of these patients expressed a distinct desire to eliminate body parts they perceived as embodying evil or being responsible for their perceived sins. This is exemplified by the relief expressed by these individuals following the completion of the act, as opposed to feelings of pain or regret [8].

In a study examining measurable variables, factors such as paranoia, auditory hallucinations, psychotic-like experiences (PLEs), and stressful life events played a role in individuals engaging in self-harming behaviors. Interestingly, the prevalence of NSSI was higher among those without PLEs than among those with them [18].

Religious delusions are a well-documented characteristic of psychotic illnesses and can become clinically significant when they lead to self-harm. Previous reports have associated schizophrenia and religious delusions with ocular injuries and auto-castration [19, 20].

In males experiencing their first episode of schizophrenia, characterized by delusions related to a body part or religious delusions, there is an elevated risk of MSM. Acute psychosis, particularly in the context of the first episode of schizophrenia, appears to be a major contributing factor to MSM. Recent reviews of self-inflicted eye injuries have revealed that nearly all serious cases stem from schizophrenia spectrum psychosis, with half of the injuries resulting in permanent vision loss during the first episode of psychosis [18].

The most prevalent indicators and risk factors for suicide in individuals with schizophrenia include a history of suicide attempts and self-harm [21]. Additionally, some studies have shown that approximately 50% of completed suicides involve a history of NSSI [18]. It is worth noticing that NSSI has been found to be a gateway to suicide in young adults, as per Whitlock, Janis et al.. Thus, it should be highlighted that enhancing and building positive relationships with parents could be beneficial for young adults with an NSSI history [22]. In the Multicenter Study of Self-Harm in England, 0.5% of adults died by suicide within 1 year of a self-harm event (0.82% of males; 0.27% of females), corresponding to an approximately 49-fold increased risk [23]. These data substantially underscore the markedly elevated suicide risk in adults following self-harm.

Patients diagnosed with schizophrenia and a history of both suicide attempts and nonsuicidal self-harm represent distinct subgroups. Compared to individuals with schizophrenia, they tend to have experienced higher levels of childhood trauma, early onset of psychotic symptoms, significant delays in receiving treatment, a heightened likelihood of recurrent suicidal behavior, and increased levels of current depressive and impulsive aggressive symptoms [24].

Finally, in the historical case of Vincent Van Gogh, although self-mutilation (specifically the cutting of his ear) has often been interpreted as evidence of psychosis, contemporary scholarship cautions against retroactively diagnosing a single psychiatric condition. Several alternative hypotheses have been proposed, including bipolar disorder, temporal lobe epilepsy, and alcohol-induced mood disorders, each with varying degrees of supporting evidence [25]. Moreover, Van Gogh's preserved insight and artistic productivity during periods around the incident raise questions about whether he was in a full psychotic state at the time. Thus, while psychosis might be one explanatory framework, it should not be assumed to be the sole or definitive cause of his self-harming behavior. Our case's diagnostic intricacy is not an isolated instance; previous attempts to retrospectively diagnose Vincent van Gogh have suggested various conditions, including temporal lobe epilepsy, bipolar disorder, and major depressive disorder, yet there is no agreement on the precise nature of his ailment. Recent studies emphasize that Van Gogh's situation illustrates the challenges in reaching a conclusive psychiatric diagnosis, especially when symptoms overlap and comorbidities exist [26]. In a similar vein, our patient exhibited a range of symptoms that defied straightforward classification, highlighting the importance of a detailed, long-term approach to both diagnosis and treatment.

In this reported case, given the patient's delusions and self-harming behavior, a treatment plan focusing on antipsychotic medication was initiated, aimed at reducing psychotic symptoms and improving mood, thereby addressing both the schizoaffective and self-harm aspects of his condition. Early and thorough diagnostic assessments of psychotic symptoms in individuals with a history of both suicide attempts and nonsuicidal self-harm are essential to prevent delayed treatment and potentially reduce the risk of recurrent nonsuicidal self-harm and severe suicide attempts in the future.

Individuals who engage in self-harm need a thorough, multitiered strategy to avert suicide. Initially, healthcare providers should focus on conducting detailed suicide risk evaluations, creating collaborative safety plans, and limiting access to lethal means. In the medium term, it is essential to enhance the treatment of any underlying mental health conditions and incorporate evidence-based therapies like dialectical behavior therapy (DBT) and cognitive behavioral therapy for suicide prevention (CBT-SP). Ensuring continuity of care is vital, especially during the vulnerable period following discharge, by providing prompt follow-up and integrating community support. For long-term management, it is important to include family psychoeducation, activate social support networks, and establish system-level pathways that facilitate monitoring and proactive engagement with individuals at high risk. Although there is strong evidence supporting these interventions, practical challenges such as resource limitations, stigma, and cultural factors must be considered when customizing these strategies.

## 4. Conclusion

Intentional self-harm, often referred to as DSH, is a strong predictor of suicide risk in schizophrenia. The tragic demise of Vincent Van Gogh, who passed away on July 29, 1890, at the age of 37, following a suicide attempt involving a firearm, a year after the notorious incident of self-inflicted ear mutilation, stands as a poignant illustration of how individuals with a history of MSM are at a heightened risk for suicide. This underscores the pressing need for clinicians to possess accurate knowledge of the risk factors associated with DSH among individuals with schizophrenia. This knowledge is crucial for identifying those at elevated risk and, whenever feasible, implementing appropriate strategies to reduce harm. Patients with schizophrenia exhibiting these risk factors necessitate vigilant follow-up and monitoring, along with the treatment of any concurrent conditions, such as depression or substance abuse. Furthermore, there is a need for increased research efforts aimed at identifying the specific risk factors for DSH in individuals with psychotic disorders and establishing the most effective measures to prevent such outcomes.

## Figures and Tables

**Table 1:** Mental status examination.

Aspect of mental status examination	Findings
General appearance and behavior	- Middle-aged man, left hand in a cast, well-kempt, poor eye contact, smiling inappropriately, shy, normal posture, uncooperative attitude

Speech	- Monotonous quality
	- Relatively long pauses between answers
	- Speech is sometimes irrelevant

Affect	- Blunt with a restricted range

Mood	- Depressed
	- Decreased reactivity and communicability

Thought process	- Slowing thoughts
	- No formal thought disorder detected in the serial mental examination

Psychotic symptoms	- Delusions of control
	- Religious delusions
	- Delusions of guilt
	- Second and third-person auditory hallucinations
	- Belief in erasing sins and seeking forgiveness from god
	- Feeling of being controlled to self-harm in response to the voices of friends and family

Judgment and insight	- Impaired

Cognition	- Oriented to time, place, and person

## Data Availability

The data that support the findings of this study are available from the corresponding author upon reasonable request.
