# Correction to “Exploring Van Gogh Syndrome: A Case Report on Schizoaffective Disorder and Self‐Harm”

**DOI:** 10.1155/crps/9865295

**Published:** 2026-02-22

S. Aly, A. A. Hasanoglu, and O. Abdallah, “Exploring Van Gogh Syndrome: A Case Report on Schizoaffective Disorder and Self‐Harm,” *Case Reports in Psychiatry* 2025 (2025): 9655675, https://doi.org/10.1155/crps/9655675.

In the article titled, “Exploring Van Gogh Syndrome: A Case Report on Schizoaffective Disorder and Self‐Harm,” information is missing in the Funding section. The corrected section appears below:

**Funding**

The funding for this publication is supported by MRC, HMC. The authors acknowledge the MRC for their financial support.

We apologize for this error.

